# Debate: Can and should psychiatrists use online information?

**DOI:** 10.1192/pb.bp.114.049973

**Published:** 2015-12

**Authors:** Elly O'Brien, Christopher Pell

**Affiliations:** Bazian Ltd, Email: elly_obrien@hotmail.com; NHS Tayside, Email: chris.pell@nhs.net

## Yes

When considering professional use of the internet, the focus tends to be on access to information. Yet the development of Web 2.0 and the growth of social media have transformed the internet from a largely read-only medium to one that facilitates interaction and user-created content. I will discuss some of the positive effects that online resources can have on professional practice, looking not just at access to information, but what we do with that information and how we interact online with fellow professionals and the public.

Doctors can access the best evidence online during consultations: using Google Scholar to identify relevant journal articles, consulting guidance using the NICE app^1^ or checking doses and contraindications on the BNF mobile app (even when you have no mobile signal). Sites that aggregate high-quality content from elsewhere, such as NICE Evidence Search (www.evidence.nhs.uk) and Trip (www.tripdatabase.com), make searching for information and staying up to date quicker and easier. Trip's ‘rapid review’^2^ even allows you to search for primary studies for an intervention in a particular population, then compares the results to provide an overall score (although not designed to replace a full systematic review, it provides a useful and rapid overview of the evidence in an area).

The potential for variation in the quality of online information is often cited as a criticism of it. Using high-quality resources helps to reduce this risk, but the principles of critical appraisal apply to any information, regardless of the medium. Wikipedia has been shown to be as reliable as traditional textbooks in psychiatry,^3^ and ‘comes close to’ *Encyclopaedia Britannica* in accuracy.^4^ Wikipedia should not be written off as a resource in research, but its contents must be subject to scrutiny and facts always cross-checked.

Online patient information is useful for patients, who can read it in their own time and refer back to it if they have queries. NHS Choices^5^ offers a wealth of information for patients, and NHS England's Information Standard^6^ helps psychiatrists to identify high-quality patient information from a variety of sources (including the Royal College of Psychiatrists),^7^ reducing the need to produce leaflets in-house. Material with the Information Standard is also included in NICE Evidence Search – so doctors can retrieve high-quality patient information alongside clinical evidence.

Blogs can be used by patients much like a traditional diary, to express themselves and track the course of their illness. Psychiatrists can also direct patients to blogs that they may find useful to read. However, there is huge variation in blogs, and are prone to a type of response bias whereby only those with the most extreme experiences (whether positive or negative) will blog about them; therefore, blogs should be carefully vetted before being recommended to patients.

Educational and informative materials can also be shared and viewed online as videos via YouTube (see the Royal College of Psychiatrists' YouTube channel^8^) or presentations on SlideShare (www.slideshare.net). Following the ‘do once and share’ principle, this gives material greater reach as well as encouraging discussion and collaboration between professionals.

Twitter offers a forum for current awareness through discovering and sharing new evidence and useful resources. It also provides a medium for interactions between professionals, where they can discuss issues and, potentially, crowdsource solutions. The Department of Psychiatry at the University of Toronto runs the International Psychiatry Twitter Journal Club,^9^ allowing a large number of professionals from around the world to discuss a paper. Those who cannot attend can catch up or continue the conversation using the hashtag #psychjc. Conferences also increasingly promote tweeting, allowing those unable to attend in person to keep up with proceedings by monitoring the relevant hashtag. Storify is a free tool that can be used to draw together various media such as photos, videos and tweets from conferences (or anything with a hashtag) to produce a record of the conference.^10^

Twitter also allows interaction beyond fellow professionals; for example, the successful Twitter campaign last year that forced major supermarkets to stop selling ‘mental health patient’ Halloween costumes.^11^ The campaign involved thousands of messages from members of the public, professionals, politicians and charities. Twitter also provides a valuable medium for promoting events such as World Mental Health Day, aimed at breaking down stigma, addressing misconceptions, advocating for patients' rights and giving patients a voice.

There is a lot of scaremongering about social media for doctors. However, arguably this is simply a new medium in which age-old problems can arise. Indeed, the General Medical Council (GMC) guidance states that: ‘The standards expected of doctors do not change because they are communicating through social media rather than face to face or through other traditional media’.^12^ So principles of patient confidentiality, respect for colleagues and maintaining appropriate boundaries with patients apply equally online and face to face. Accepting ‘friend requests’ and ‘follows’ from patients or caregivers requires careful consideration, especially with vulnerable patients. But how different is this to psychiatrists having to decide how to deal with bumping into a patient at the supermarket? Social media can blur the distinction between the professional and the personal, but there are solutions, such as separating these personas into different accounts and increasing privacy settings (for example, having a protected account on Twitter). What should not be overlooked is the potential of social media for enabling interaction with patients and their caregivers, for new dialogues that challenge professionals' perceptions of service users' experiences and that create a more collaborative care model.

Psychiatrists must always question the information in front of them and the source from which it originates, regardless of the medium. Online resources enable psychiatrists to keep up to date with the latest research and to engage with it in a more interactive way. Psychiatrists now also have the opportunity to create and share their own high-quality media to raise the profile of the profession, and to combat misinformation and stigma. The use of online resources should not only be encouraged but be considered essential in contemporary psychiatry.

## No

Social media and Web 2.0 are driving major social changes – one seventh of humanity has signed up for a Facebook account since its inception just a decade ago – and it would be naive to think that psychiatry would ignore the many advantages these changes bring.^13^ More than ever before we have access to the best-quality information, opportunities for professional and public collaboration and engagement, and multiple forums in which to raise awareness and standards in psychiatry. Or so it would seem.

In reality, our ability to use these technologies in professional practice has become fraught with difficulty as psychiatrists try to adapt to this shifting landscape. Indeed, new avenues for problems seem to crop up with alarming regularity, whether it be emerging legal trends, fresh ways to be censured by the GMC, or even novel clinical presentations such as addictive behaviour towards new technology.^14^ Such was the concern about professional use of the internet that the GMC update to *Good Medical Practice* now clearly defines the expected standards of practice that UK doctors use when going online.^12^

Whether we can access professional information online is often dependent on what is being searched for, and the location of the clinician. Many of the internet resources we take for granted at home are blocked or limited by internet filters. Websense and similar filters err on the side of caution to protect National Health Service networks from malware at the expense of many sharing and networking sites, rendering useful resources such as Dropbox, Slide-Share, Twitter and YouTube all but inaccessible from the hospital. Often, lack of hardware or mobile data access within the hospital can make accessing information online a frustrating exercise. Much more needs to be done to provide clinicians with smart devices and to open up wireless internet access within healthcare settings before we are truly able to take advantage of online information.

Even assuming this is achievable, there is an emerging opinion that doctors spend too much time staring at a computer screen and typing, rather than engaging with, actively listening to and carefully thinking about their patients. Furthermore, the illusion that such technologies improve our workflows by allowing us to multitask (for example, by searching for pertinent information during interviews or meetings) is severely challenged by the finding that our cognitive abilities and working memory are limited. The simple fact is that multitasking makes us more distraction prone – so we perform multiple tasks with an increasing lack of attention and efficiency.^16,17^

Let us assume, though, that you have relatively unfettered access to the internet and have easy access to a computer in a distraction-free environment: should you use the internet to find professional information?

An initial problem is quality control. In the ‘information age’ critical appraisal is more than ever a vital skill, particularly with the proliferation of open access online journals with seemingly less-than-robust peer-review structures to safeguard article accuracy.^18^ This takes up time that you may not have, yet fails to provide the same level of coverage as a systematic literature search.^19^ Although we like to think that we can sift out the incorrect information, we are all prone to inherent biases when analysing multiple sources of data. Interrogating Google or other search engines for clinical information may compound this by selectively presenting data according to the search engine's own algorithms, rather than by the robustness of studies themselves.

The issue of quality of information costs us more time, as those attending our clinics and hospitals may now come armed with information they have uncovered online regarding their symptoms and treatments. In each case the validity and relevance of the information must be examined, before explaining to the individual why the ‘facts’ they have found may not be quite as they seem. This also extends to information that a patient may have learned online about their doctor, either through rating sites or informally via a Google search. Social media has considerably blurred the boundaries between our professional and private personas. While some professionals strive to separate these two aspects of their lives online, this is hard to achieve fully in practice. Psychiatry is no stranger to boundary issues, however. As our patients and their carers enter cyberspace, online interactions require care and attention in order to avoid difficulties in subsequent clinical interactions.

Although doctors are generally becoming more experienced at safely managing their digital identities, many still do not fully understand or adjust privacy settings on social media sites. Still other doctors fall foul of expected professional standards in terms of what information they make available online.^20,21^ With the increasing integration of location-sharing functions to such sites, this opens up new avenues for access and contact not previously available and may lead to unwanted communication or harassment. Some go as far as to suggest that professionals may wish to limit their use of social media so as to reduce the risk of stalking.^22^ All of this places considerable onus on psychiatrists to be at least aware of their digital footprint; better still, to actively curate their web presence and privacy settings so that oversharing and misinformation are swiftly identified and tackled. This same challenge extends to the specialty as a whole, as any search of Google or YouTube for terms such as ‘ECT’ or ‘psychiatry’ can yield reams of misinformation.

And what of reciprocity? Can patients expect to have their online identities scrutinised by healthcare staff prior to attending clinics, or should this information (however publicly available) require their explicit consent before being used in clinical decision-making?

It is questions such as this that highlight the rapidity with which these technologies have changed the landscape of interpersonal interactions within our society. With a mere decade of experience and with new social media trends and websites constantly emerging, no-one yet has a clear idea where the ethical, legal and professional sensibilities will eventually settle. What is clear is that using online information will necessarily change our practice, both by requiring greater attention to and scrutiny of the information yielded, and by changing the parameters of the doctor-patient relationship.

With our seeming acceptance of the erosion of personal privacy and our constant searching, editing and sharing of information, we may unwittingly be setting up as yet unknown difficulties and challenges for professional practice in the future. Whether we can skillfully navigate the minefields of professional information in the online world remains to be seen.
